# YouTube as a Patient Information Source for Tendon Repair Surgery

**DOI:** 10.7759/cureus.43890

**Published:** 2023-08-21

**Authors:** Shubham Mishra, Romil H Maniar, Britney Moody, Nadia Djahanshahi, Yanisa Sompornboriboon, Keval B Patel

**Affiliations:** 1 Surgery, The University of Edinburgh, Edinburgh, GBR; 2 Surgery, K.J. Somaiya Hospital, Mumbai, IND; 3 Surgery, St. George’s University, True Blue, GRD; 4 Surgery, Khon Kaen University, Khon Kaen, THA; 5 Surgery, Narendra Modi Medical College, Ahmedabad, IND

**Keywords:** tendon surgery, quality assessment, surgery, tendon repair, youtube®

## Abstract

Background

For tendon rupture, a disabling musculoskeletal injury, surgical management is considered the gold standard. The goal is to reduce complications and enable the patient to regain full mobility and strength. With the rise of modern internet accessibility and social media, YouTube has become a frequently used platform for all ages; however, the accuracy and reliability of the videos and the information therein may be a point of concern.

Methodology

This cross-sectional, observational study was designed to analyze tendon repair surgery information available on YouTube. Data were collected using a Google Forms questionnaire which included questions analyzing the videos and their content and the professional status of the uploaders. Quality and reliability scores were assessed through the Global Quality Score (GQS), reliability score, and Video Power Index (VPI). Statistical analysis was performed using SPSS software (IBM Corp., Armonk, NY, USA). We included videos one minute to twenty minutes in length that were relevant to the topic of tendon repair surgery and in the English language.

Results

A total of 82 videos were evaluated, of which 67 videos were chosen. A comparison of GQS, reliability score, and VPI based on the type of uploader was done using the Kruskal-Wallis test. The association between GQS and reliability score with that of the video uploader was found to be statistically significant (p < 0.05). Videos uploaded by hospitals had the highest GQS (4) and reliability score (4).

Conclusions

On comparing the uploader type, videos uploaded by hospitals had good quality and were useful for patients. The quality and reliability of the videos were almost above average. Only half discussed the signs and symptoms and even fewer discussed pre- and post-procedural care. The most important aspect, i.e., prevention, was mentioned in none of the videos. Due to our limitations, expanding the number of accounts used for search and increasing the number of videos might overcome the search algorithm.

## Introduction

Tendons are strong structures composed of tenocytes, chondrocytes, and synovial cells as well as a predominant extracellular matrix containing type I collagen, elastin, glycoproteins, and proteoglycans [1,2]. These components form fibrils, fibers, and fascicles creating tendons that anchor the muscle to bone [1,2]. Of the possible musculoskeletal injuries, tendon injuries are the most common, with Achilles and patellar tendons being the most common site of injury [1]. These injuries occur due to degenerative disease, aging, athletic activities, penetrating trauma, and overuse [1,3].

Tendon injuries can be repaired via non-surgical, reparative, and regenerative treatments [1]. While the type of management employed remains a decision made by patients and their physicians, surgical management is considered the gold standard for athletes and younger patients [3]. Surgical options include grafts (autografts, allografts, and xenografts), tissue engineering (biodegradable polymers such as silk protein, collagen, and hyaluronic acid), and prosthesis [1-3]. Regardless of the chosen surgical management, the goal is to reduce complications such as re-rupture and damage to the surrounding soft tissue and nerves and to return the patient to full mobility and strength [3].

With the rise of modern internet accessibility and social media, YouTube has become a frequently used platform for all ages drawing upwards of two billion views per day [4,5]. While YouTube is used for many purposes, more and more people are using it for educational purposes, with 33% of patients stating they have used YouTube for medical education according to a 2018 national health survey [4]. The video format in which information is presented on YouTube is proving to be a strong educational tool for both patients and healthcare workers in addition to text resources [4,5].

YouTube’s expansive topics and widespread accessibility make it a valuable resource for patients seeking information about specific ailments and medical conditions. However, the accuracy and reliability of these videos and the information therein may be a point of concern. Therefore, this study aimed to access the quality and accuracy of available information to allow for the expansion of accurate and accessible information for patients about tendon repair surgery.

## Materials and methods

This cross-sectional, observational study was designed to analyze information regarding tendon repair surgery available on YouTube. The study was conducted on April 20th, 2023, utilizing a Google Forms-based questionnaire that was created and pre-tested using 10 videos. A total of six medical researchers utilized the questionnaire to collect data and analyze YouTube videos using the keyword “tendon repair surgery.”

YouTube videos on tendon repair surgery published in English and with a video length ranging between one and twenty minutes were included in the study. The questionnaire included questions about the time frame, total views, likes, comments, and quality of information; whether the videos included information such as symptoms, etiology, investigations, treatment, rehabilitation, mortality, support groups, and shared experiences; and if there was a promotional aspect to the video. In addition, the questionnaire also asked about the professional status of the uploaders categorizing them as medical doctors, hospitals, healthcare organizations, news agencies, patients, and other entities. The responses were recorded in Google Sheets and transferred to Microsoft Excel.

Quality and reliability scores were assessed using the Global Quality Score (GQS), reliability score, and Video Power Index (VPI). Statistical analysis was performed using SPSS software (IBM Corp., Armonk, NY, USA).

The GQS is a measure used to assess the overall quality and performance of video content. It takes into account various factors such as video resolution, clarity, audio quality, and overall production value. Higher GQS values indicate better quality and more engaging video content. The reliability score is a metric that evaluates the credibility and trustworthiness of video content. It assesses factors such as accuracy of information, sourcing, fact-checking, and adherence to journalistic standards. The reliability score helps viewers gauge the credibility of the video and make informed decisions regarding the information presented.

The VPI measures the overall performance and influence of a video across various platforms. It considers factors such as viewership, engagement metrics (likes, shares, comments), social media impact, and overall reach. VPI helps assess the popularity, effectiveness, and potential viral impact of a video. By analyzing these scores, content creators can improve the quality of their videos, advertisers can gauge the effectiveness of their campaigns, and viewers can make informed choices about the content they consume [6-8].

As the study did not involve any human participants, institutional review board approval was not required.

## Results

A total of 82 videos were evaluated, of which 67 met the inclusion criteria. Table 1 shows the characteristics of the analyzed videos.

**Table 1:** Characteristics of the YouTube videos analyzed.

Time since upload	
Less than one month (<30 days old)	5 (7.5%)
More than a month to one year (31–365 days old)	7 (10.4%)
More than one year (>365 days)	55 (82.1%)
Popularity	
Total number of views	24,792,179
Total number of likes	157,379
Total number of dislikes	5,398
Total number of comments	12,367
Type of uploader	
Doctor	36 (53.7%)
Hospital	17 (25.4%)
Healthcare organization/News/Patient/Other	14 (20.9%)

The 67 videos analyzed received a total of 24,792,179 views, with 157,379 likes, 5,398 dislikes, and 12,367 comments. The majority of the videos (82.1%) were more than a year old. It was established that 53.7% of the videos were published by doctors, whereas 25.4% were uploaded by hospitals. Furthermore, 20.9% of the videos came from healthcare organizations, news outlets, patients, or other relevant groups. Table 2 presents an in-depth breakdown of the information disseminated in the analyzed videos.

**Table 2:** Information about tendon repair surgery in the YouTube videos.

Information	Number of videos (%)
Description of symptoms	34 (50.75%)
Information about the cause/etiology of tear	29 (43.28%)
Information about investigations/tests	25 (37.31%)
Information about prevention/vaccines	0 (0%)
Information about treatment	62 (92.54%)
Information about rehabilitation	27 (40.3%)
Information about people/patients sharing their experience	7 (10.45%)
Promotional content by pharmaceutical companies or by doctors	3 (4.48%)
Description of the reason for surgery	39 (58.21%)
Description of the anatomy of the involved area	53 (79.1%)
Information about the pre-procedural care/preparation phase	19 (28.36%)
Information about post-surgical care?	25 (37.31%)
Information about prognosis after surgery	20 (29.85%)

Overall, 92.54% of the videos had information about the treatment and surgery to be performed. Furthermore, 79.1% of the videos described the anatomy of the involved area, 58.21% described the reason for surgery, 29.85% provided the prognosis after surgery, 10.45% had patients sharing their own experience, and only 4.48% of videos had promotional content by pharmaceutical companies or doctors. Table 3 shows the comparison of GQS, reliability score, and VPI based on the type of uploader.

**Table 3:** Comparison of GQS, reliability score, and VPI based on the type of uploader. GQS = Global Quality Score; VPI = Video Power Index

	Doctors (n = 36)	Hospital (n = 17)	Healthcare organization/News/Patient/Other (n = 14)	P-value and test used
	Median (IQ1, IQ3)	Median (IQ1, IQ3)	Median (IQ1, IQ3)	Test used: Kruskal-Wallis test
VPI	21.74 (5.22, 53.47)	31.32 (5.50, 57.47)	65.79 (10.81, 590.42)	P-value = 0.324
GQS	3 (2, 3)	4 (3, 4.5)	3 (3, 4)	P-value = 0.003
Reliability score	3 (3, 4)	4 (3, 4.5)	3 (3, 4)	P-value = 0. 032

To compare these, the Kruskal-Wallis test was used. On analyzing the relationship between VPI and the type of uploader, scores were found to be statistically insignificant (p > 0.05). The association between GQS and reliability score with that of the video uploader was found to be statistically significant (p < 0.05). Videos uploaded by hospitals had higher GQS (4) and reliability scores (4) compared to the videos uploaded by doctors or healthcare organizations.

## Discussion

This study aimed to examine and assess the most relevant videos for the search term “tendon repair surgery” on YouTube with regard to competence, quality, and dependability.

We found that the quality and reliability of YouTube videos about tendon rupture surgery were almost above average. Patients are increasingly turning to social media to learn more about their medical concerns, and YouTube is home to a sizeable collection of videos with a medical focus. Despite the fact that these videos are very popular, the material and video quality seem to be on par. Our findings differed from other studies examining YouTube videos about rotator cuff tears, lower extremity injuries, distal biceps tendon ruptures, and arthroplasty, which suggested that these videos could be subpar patient education tools based on GQS JAMA criteria and DISCERN score [9-11]. According to a recent YouTube analysis of distal biceps tendon rupture [9], the videos submitted by academic sources did not appear to be of a higher caliber than those uploaded by non-academic sources. Our results differ from those of Brian et al. as tendon repair surgery videos posted by academic sources received higher content or quality ratings than those from non-academic sources [9].

Other similar studies, such as by Keelan et al. [12], first assessed the quality of the immunization-related YouTube videos and discovered low-quality scores for various medical disorders. The dependability score for rotator cuff surgery videos in another study was 0.58 on average.

The reliability score, or GQS, and the average number of views, the number of likes, and the VPI of all videos were also not shown to be statistically significantly correlated in this study. Video dislikes were described as determinants of YouTube video dependability in a previous study on the meniscus, but this was not the case in this study where we only used the reliability, GQS, and VPI scores as determinants [13].

According to Erdem et al., the video source is the most crucial element in obtaining adequate information [14]. In this study, doctors made up the majority of the sources (53.7%), followed by hospitals (25.4%). Despite having higher quality ratings than other groups, videos produced by hospitals were far from providing sufficient high-quality information. Only about half (50.75%) of the videos used in this study discussed the signs and symptoms of tendon rupture, even fewer (43.28%) discussed the cause or etiology of tendon rupture or tear, and even fewer (37.31%) discussed the required investigation and testing that must be performed.

The numerous treatment options that are available for different types and degrees of tendon rupture were discussed in almost all of the videos (92.54%). For the viewers’ better understanding, the majority of the videos (79.1%) detailed the affected area’s anatomy. More than half (58.21%) of the videos explained the rationale behind the procedure.

A little over a quarter of the videos provided information about pre-procedural care, post-surgical care, and the prognosis after surgery (28.3%, 37.31%, and 29.85%, respectively).

None of the videos gave any information about the prevention of tendon rupture, mortality involving the disease, and information about support groups. Videos containing the above information are very useful for the vast majority of viewers as it helps them alleviate any kind of fear regarding the surgery and allows them to connect with people with similar complaints or those who have undergone a similar surgery in the past.

Erdem et al. were the first to describe the VPI, which was used to evaluate the effectiveness of videos by counting views and likes [14]. The most popular groups in this study with greater VPI were commercial and patient-based videos. However, both the GQS and the reliability score had a negative correlation with VPI.

These findings show that most YouTube users were interested in videos containing insufficient information about the surgery rather than videos containing step-by-step details of the surgery. Numerous factors may be connected to these findings. First, viewers of the doctor-submitted YouTube videos might not understand them. Second, the majority of YouTube users are not medical professionals and do not have the same expectations from the information that doctors supply. Similar findings were reported by Ferhatoglu et al. [15], who found a negative relationship between the popularity of the videos and video quality ratings. The length of the videos could be another factor. Biggs et al. found that shorter videos had more views than longer videos [16].

Patient decision-making may be impacted by cognitive bias, particularly the anchoring effect that describes the common human tendency to rely too heavily on the first piece of information offered. These beliefs can be reinforced by online information sources, similar to what is seen in patients with rotator cuff tears (the notion that the patient has a tear and that it needs to be fixed). In the end, the surgeon’s job is to help patients who have misconceptions about their injury or the treatment options redirect themselves; unfortunately, this can be more challenging in the face of inaccurate online information.

Strengths and limitations

This study conducted a comprehensive assessment of YouTube videos related to tendon repair surgery. It evaluated factors such as quality, reliability, and content coverage and provides a holistic analysis of the available information. Furthermore, using three different scoring criteria (GQS, reliability score, and VPI) to rate the content of the videos increases the quality of the study findings. This study also identifies the gaps in content coverage, allowing for the future development of more comprehensive and informative content for patients.

However, there are several limitations. First, the data collection was done by searching for videos based on popularity. In doing so, it is possible that we may have missed less popular but good-quality videos. Another limitation can be due, in part, to YouTube’s search algorithm. By using user age, gender, geolocation, and watch history to personalize search results, YouTube users may be accessing more popular videos that the algorithm shows them, not necessarily the highest-quality videos.

Second, because YouTube video measures such as the number of views and likes are updated often, the study data are only accurate as of the search date. Third, the dependability score, GQS, and VPI assessment scoring systems that we utilized are subjective and unvalidated. The dependability score criteria may not apply to YouTube videos because they were created to evaluate medical information on websites rather than in videos. Because many of the videos failed to meet certain reliability scoring requirements, some criteria may have a greater impact on the final score than others; as a result, not all criteria are equally important.

Fourth, we did not evaluate the readability of these videos in our analysis. Additionally, we did not evaluate the intended target demographic for the video. As patients were not the target audience, it is likely that surgical procedure videos uploaded for surgeon training may have poorer quality and dependability scores in comparison to patients. Future research could examine the connection between pre-consultation internet knowledge, cognitive bias, and decision-making. Future research should also systematically evaluate where patients look for educational resources at home.

Fifth, the GQS and the reliability scores were given by different people for different videos which can lead to variation in interpretation and scoring.

## Conclusions

On comparing the type of uploaders, videos uploaded by hospitals have good quality and are useful for patients. However, there was no difference in the VPI between those uploaders. The quality and reliability of the videos were almost above average, but only half discussed the signs and symptoms, and even fewer discussed pre- and post-procedural care.

The most important aspect, prevention, was mentioned in none of the videos. Due to our limitations, expanding the number of accounts used for search and increasing the number of videos might overcome the search algorithm.
